# A Heat Shock Transcription Factor *TrHSFB2a* of White Clover Negatively Regulates Drought, Heat and Salt Stress Tolerance in Transgenic *Arabidopsis*

**DOI:** 10.3390/ijms232112769

**Published:** 2022-10-23

**Authors:** Muhammad Zafar Iqbal, Tong Jia, Tao Tang, Muhammad Anwar, Asif Ali, Muhammad Jawad Hassan, Youzhi Zhang, Qilin Tang, Yan Peng

**Affiliations:** 1 College of Grassland Science and Technology, Sichuan Agricultural University, Chengdu 611130, China; 2 Guangdong Key Laboratory of Plant Epigenetics, College of Life Sciences and Oceanography, Shenzhen University, Shenzhen 518055, China; 3 Key Laboratory of Southwest Crop Genetic Resources and Genetic Improvement, Ministry of Education, Rice Research Institute, Sichuan Agricultural University, Chengdu 611130, China; 4 Maize Research Institute, Sichuan Agricultural University, Chengdu 611130, China

**Keywords:** *Trifolium repens*, heat shock factor, abiotic stress, overexpression, gene silencing

## Abstract

Heat shock transcription factors (HSF) are divided into classes A, B and C. Class A transcription factors are generally recognized as transcriptional activators, while functional characterization of class B and C heat shock transcription factors have not been fully developed in most plant species. We isolated and characterized a novel HSF transcription factor gene, *TrHSFB2a* (a class B HSF) gene, from the drought stress-sensitive forage crop species, white clover (*Trifolium repens*). *TrHSFB2a* was highly homologous to *MtHSFB2b*, *CarHSFB2a*, *AtHSFB2b* and *AtHSFB2a.* The expression of *TrHSFB2a* was strongly induced by drought (*PEG6000* 15% *w*/*v*), high temperature (35 °C) and salt stresses (200 mM L^−1^ NaCl) in white clover, while subcellular localization analysis showed that it is a nuclear protein. Overexpression of the white clover gene *TrHSFB2a* in *Arabidopsis* significantly reduced fresh and dry weight, relative water contents (RWC), maximum photosynthesis efficiency (Fv/Fm) and performance index on the absorption basis (PI_ABS_), while it promoted leaf senescence, relative electrical conductivity (REC) and the contents of malondialdehyde (MDA) compared to a wild type under drought, heat and salt stress conditions of *Arabidopsis* plants. The silencing of its native homolog (*AtHSFB2a*) by RNA interference in *Arabidopsis thaliana* showed opposite trends by significantly increasing fresh and dry weights, RWC, maximum photosynthesis efficiency (Fv/Fm) and performance index on the absorption basis (PI_ABS_) and reducing REC and MDA contents under drought, heat and salt stress conditions compared to wild type *Arabidopsis* plants. These phenotypic and physiological indicators suggested that the *TrHSFB2a* of white clover functions as a negative regulator of heat, salt and drought tolerance. The bioinformatics analysis showed that *TrHSFB2a* contained the core B3 repression domain (BRD) that has been reported as a repressor activator domain in other plant species that might repress the activation of the heat shock-inducible genes required in the stress tolerance process in plants. The present study explores one of the potential causes of drought and heat sensitivity in white clover that can be overcome to some extent by silencing the *TrHSFB2a* gene in white clover.

## 1. Introduction

White clover, belonging to the genus *Trifolium*, is an excellent worldwide cultivated forage due to its high-yield characteristics, better forage quality and strong biological nitrogen fixation ability. It is an integral part of the grass industry. The development of white clover can promote grain conversion to feed and encourage grass and animal products in competitive markets. However, it is vulnerable to drought and heat stresses and shows significant growth retardation under stress conditions [1], which restrict its development and production globally [2,3]. Therefore, it is of great theoretical and practical significance to strengthen the research to improve drought tolerance in white clover and cultivate drought-tolerant varieties by identifying, studying and manipulating downstream genes and molecular mechanisms.

Heat shock proteins (HSPs), as molecular chaperones, play significant roles in responding to environmental stresses and taking part in protein folding, assembly and stability, which are important threats under stressed conditions in plants [4,5]. The expression of HSPs is controlled by heat shock transcription factors, containing conserved binding domains known as heat stress elements (HSE), mostly existing in promoter regions of the heat stress-inducible/responsive genes and are conserved almost in all eukaryotes. Structurally heat shock transcription factors (HSFs) comprise of a main DNA-binding domain (DBD) of a helix-turn-helix motif in the N-terminal region, which generally binds with the HSE present in the promoter region of heat stress-inducible genes [6]. Following the DBD domain, there is an oligomerization domain (OD), associated with heptad hydrophobic repeat motifs, and a NLS (Nuclear localization signals) domain containing a basic amino acid residue motif and a leucine-rich motif in the HR-C region for export (NES). The C-terminal region is comparatively less conserved, comprising of hydrophobic, aromatic and acid amino acid (AHA) motifs, which are essential for transcriptional activity [6]. Since the first plant HSF isolation from *Solanum lycopersicum* in the 1990s [7], many HSFs have been identified and characterized [8]. For instance, there are 21 HSFs in *Arabidopsis*, 25 in *Oryza sative,* 31 in *Zea may* ssp. *mays*, 38 in *Glycine max* and 56 in wheat, divided into classes A, B and C based on their conserved domain structures [6,9,10,11,12,13,14,15]. Most of the previous research focused on class A HSFs, which generally regulate the transcriptional activities of the genes involved in different environmental stress responses and the seed development process [16,17,18,19,20]. The class B family members do not contain insertions into the HR-A/B domain, while class A has 21 amino acid insertions and class C contains 7 amino acid insertions and class B HSFs generally lack the AHA motif in the C-terminal domain [21,22], which is essential for transcriptional activity. Thus, some Class B HSFs do not show transcriptional activity or act as the co-activator by interacting with class A HSFs and generally suppress the activation of downstream stress-inducible genes [5]. For instance, *HSFB1* of a tomato acts as a co-activator along with *HSFBA1* and modulates the expression activity of certain housekeeping and viral genes under high-temperature stress [23,24]. In *Arabidopsis*, the *HSFB1* and the *HSFB2b* have already been reported to negatively control the expression of many stress-inducible genes (HSFs), including *HSFA2*, *HSFA7a, HSFB1*, *HSFB2b* and Vin3, along with several other heat shock transcription factors [25,26]. In *Arabidopsis*, the single and double mutants of the *HSFb1* and the *HSFb2b* significantly enhance the tolerance of Arabidopsis against abiotic stress tolerance [27]. In a previous transcriptome analysis of white clover under drought stress conditions, it was perceived that a transcription factor homolog to *HSFB2* of Chickpea showed a stronger expression under drought stress [28].

The economically important forage, white clover, is vulnerable to abiotic stresses, adversely affecting its normal biological and physiological activities and interrupting fundamental cellular structures [29,30]. Therefore, it is important to continuously explore effective means and resources to improve white clover’s biotic and abiotic stress tolerance. Previously, we explored the physiological, transcriptomic and metabolomics responses of white clover under drought stress conditions and found highly differential expressions of the HSFB2 homolog [28]. To assist a strategy for the molecular improvement of white clover and broaden the understanding of how heat shock transcriptional factor and heat shock proteins modulate stress responses in white clover, we isolated and characterized a class B heat shock transcription factor HSFB2 from white clover cDNA and named it as *TrHSFB2a* based on conserved domains characteristics. The qRT-PCR method was employed to investigate the spatial and temporal expression of *TrHSFB2a* under different stress conditions. Furthermore, we synthesized overexpression *Arabidopsis* and compared it with wild-type and T-DNA lines of *Arabidopsis* in which the homolog of *TrHSFB2a* was silenced by RNA interference (RNAi) to determine how HSFB2a protein responds under drought, heat and salt stress conditions. The current study results would improve the understanding of the molecular mechanism of the responses to extreme environmental conditions in white clover and explore the regulatory role of HSFs during ecological stress responses in plants.

## 2. Results

### 2.1. TrHSFB2a Was Predicated as One of the Class B HSF Proteins

**Figure 1:**
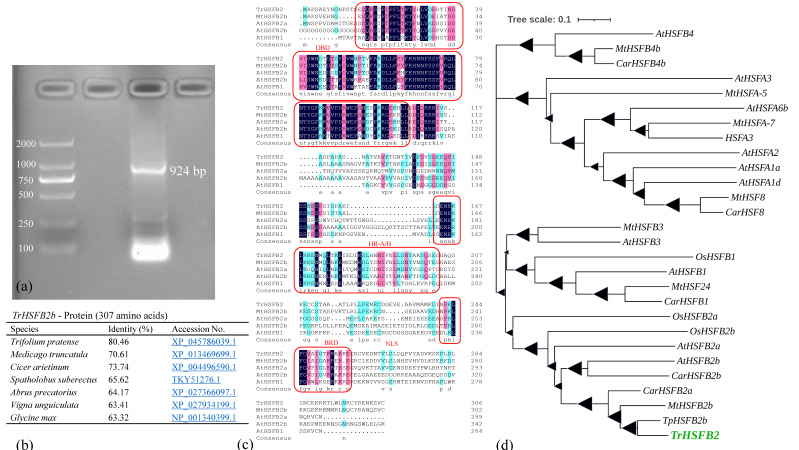
The characteristics of *TrHSFB2a* gene and protein sequence, and the phylogenetic analysis. (**a**) shows amplified DNA fragment of *TrHSFB2a* gene from white clover cDNA separated on 1.5% agarose gel, (**b**) sequence similarity of the predicted protein of *TrHSFB2a* gene with other species, (**c**) comparison of amino acids sequences of *TrHAFB2a* with related HSF proteins—highly homolog amino acids residue are shaded and conserved domains are boxed. (**d**) Phylogenetic relationship of the protein of *TrHSFB2a* gene with other plant species and other members of HSF family of the *Arabidopsis thaliana*. Triangles size in branches represents bootstrap values. Phylogenetic tree analysis was based on minimum evolution using Mega (version 11).

The sequence of the isolated *TrHSFB2a* gene comprises an open reading frame (ORF) of 924 bp (Figure 1a), which constitutes a protein of 307 amino acids (Figure 1c). Inter-protein scan results showed that this protein belonged to the heat shock transcription family and was predicated as the heat stress transcription factor B-2a (HSFB2-a). It contained a heat shock factor-type, DNA-binding domain (HSF-DNA-bd) of 93 amino acids (20–113), commonly associated with DNA-binding transcription factor activity, sequence-specific DNA binding and regulation of DNA-dependent cellular transcription. The N-terminal region contained a helix-turn-helix motif (H2-T-H3) in the center of the DNA binding domain (DND) (Figure 1c). Based on the homology of amino acids, *TrHSFB2a* showed an overall 80.46%, 70.61% and 73.74% homology with *TpHSFB2b*, *MtHSFB2b* and *CarHSFB2a*, respectively (Figure 1b). Additionally, amplification and sequencing of *TrHSFB2a* from white clover genomes showed it has one intron of 107bp, similar to other HSF genes of plants. Based on conserved domains of HSF genes, these are divided into Class A, B and C members. Phylogenetic tree constructed using 27 HSF proteins showed *TrHSFB2a* clustered with class B HSF proteins of closely related species and *Arabidopsis*, indicating that it is one of class B HSF proteins (Figure 1d).

Bioinformatics tools were used to understand physiochemical and structural charac-teristics of TrHSFB2a and results are presented in Supplementary Figure S1. Using pro-param predication [31], it was found that the molecular formula of TrHSFB2a was C1499H2383N425O475S11, molecular weight was 34,311.63, theoretical isoelectric point was 8.7, instability index was 49.92, and it was an unstable protein. DeepTMHMM [32] predicted that it was a globular protein and had no transmembrane structure (Supple-mentary Figure S1a). Hydrophobicity analysis using ProtScale revealed that it was a hy-drophobic protein (Supplementary Figure S1b), with an average hydropathicity −0.566. The secondary structure of the encoded protein predicted by SOPMA [33] showed that the secondary structure was composed of 33. 88% α helix, 2.93% β-turn, 55.70% random coil and 7.49% extended strand (Supplementary Figure S1d). The phosphorylation sites of protein sequence determined by Netphos 3.1 server [34] showed TrHSFB2a protein had 58 phosphorylation sites, including 32 serine sites, 19 threonine sites and 7 tyrosine sites (Supplementary Figure S1c). Protein signal peptide analysis using Signalp 4.1 server [35] showed that the protein had no signal peptide (Supplementary Figure S1e). The spatial protein structure predicted by SWISS-Mod. server [36] revealed that the three-dimensional structure had a monomer structure (Supplementary Figure S1f).

### 2.2. TrHSFB2a Is a Nuclear Protein

**Figure 2:**
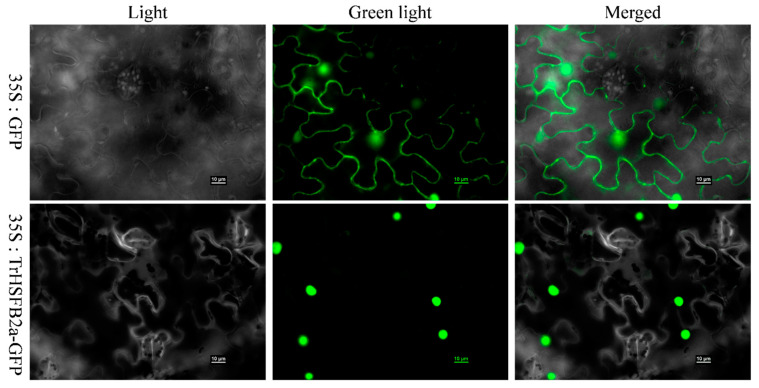
Subcellular localization of TrHSFB2a::GFP protein. The TrHSFB2a::GFP vector and an empty vector were transferred into tobacco leaves for transient expression and observed under the fluorescent microscope. Green color shows *TrHSFB2a* protein signals. Scale bar is 10 um.

*TrHSFB2a* was predicated as transcription factors, and transcription factors generally function in the nucleus. To confirm this, we initially used the WoLF PSORT program and predicted its location in the nucleus, then ligated the coding region of *TrHSFB2a* to 3′ end of GFP gene in pSUPER1300 containing 35S promoter, while GPP gene alone with promoter served as control. The subcellular location of *TrHSFB2a* protein was determined by transiently expressing TrHSFB2a::GFP fusion protein in the leaves of *Nicotiana benthamiana*. As expected, the TrHSFB2a::GFP protein is localized in the nucleus (Figure 2).

### 2.3. Relative Expression of TrHSFB2a in White Clover under Different Conditions

**Figure 3:**
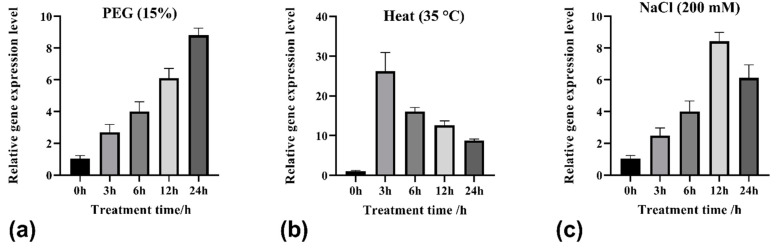
Relative gene expression pattern of *TrHSFB2a* after drought, heat and salt treatments. (**a**–**c**) Display expression level of *TrHSFB2a* under polyethylene glycol 6000 15% *w*/*v*, heat (35 °C) and salt (NaCl 200 mM) stresses, respectively, with time course 0, 3, 6, 12, and 24 h after treatments. Error bars represent the standard deviation (SD) of three independent biological replicates.

A previous transcriptome study of our research group showed that *TrHSFB2a* is strongly expressed in white clover under drought stress [28]. After isolating *TrHSFB2a* from white clover, its expression pattern was studied. The four-week-old white clover plants were subjected to drought (PEG6000 15% *w*/*v*), heat (35 °C) and salt (NaCl 200 mM) stresses. Total RNA of whole plants (root + shoot) was isolated after 0 h, 3 h, 6 h, 12 h and 24 h and used to construct cDNA for determining relative gene expression levels. A relative expression of *TrHSFB2a* was determined under normal conditions (0 h) in white clover, indicating that this gene is also expressed under normal conditions, at least in one-day-old white clover plants. Under polyethylene glycol (PEG6000 15% *w*/*v*) and salt stress of 3 h, its increased expression was observed, which kept increasing for 12 h under salt stress and, at least, up to 24 h under drought stress. However, the expression pattern of *TrHSFB2a* under heat stress was not the same. In heat stress, a strong expression of *TrHSFB2a* was detected after 3 h, and then it reduced continuously but remained relatively higher than the highest levels observed under salt and drought stresses (Figure 3). These results indicated that *TrHSFB2a* was involved in abiotic stress responses in white clover and more strongly induced under heat stress.

### 2.4. TrHSFB2a Negatively Regulates Drought Tolerance in Transgenic Overexpression Arabidopsis

**Figure 4:**
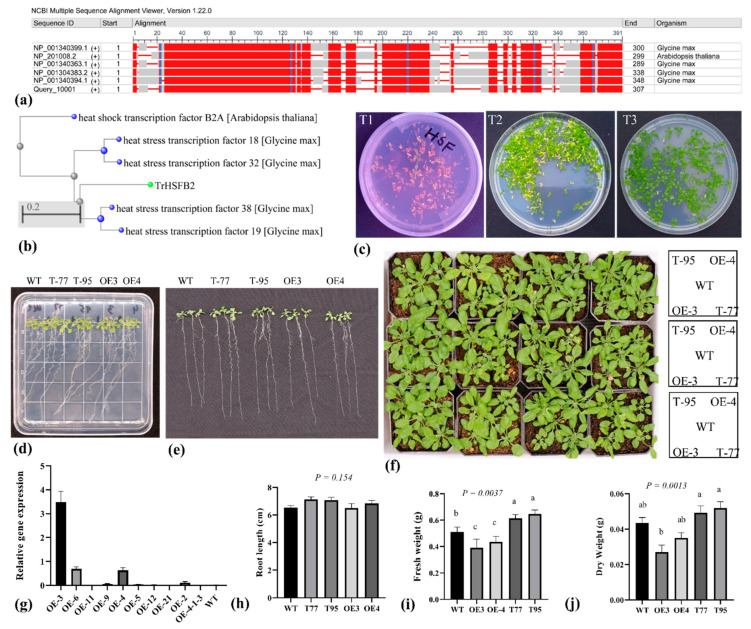
Homologous relationship of *TrHSFB2a* based on conserved domains and construction of overexpression lines of *TrHSFB2a*. (**a**) Homologous relationship of *TrHSFB2a* with most related proteins, with most conserved regions shaded by red color (**b**) Phylogenetic relationship of *TrHSFB2a* based on conserved domains, (**c**) selection of overexpression lines of *TrHSFB2a* on 1/2MS medium containing 50 mg/L Kanamycin for developing homozygous lines. Only transformed plant could grow on selection media, while non-transformed turned into yellow and died (**d**,**e**) Show plant stature and root growth of wild type (Wt), RNA interference T-DNA lines (T-77 and T-95) and overexpression lines (OE3 and OE4) grown on 1/2MS medium containing 3% sucrose and 0.7% agar. (**f**) Display the morphology of *TrHSFB2a* overexpression lines, T-DNA lines and Wt type plants in soil, (**g**) relative gene expression in overexpression lines, (**h**,**i**,**j**) display root length (cm), fresh weight (g) and dry weight (g), respectively, of wild and transgenic plants under normal growth condition. Bars represent mean ± SD values, and different small letters (a, b and c) represent significant statistical difference among means (one-way ANOVA, followed by Tukey test) between different lines.

The detection of *TrHSFB2a* expression under abiotic stress observing its role in stress responses in detail. Therefore, the overexpression vector of *TrHSFB2a* was constructed and transferred into *Arabidopsis thaliana*, and thereby, constructed the *TrHSFB2a* overexpression Arabidopsis. After developing homozygous T3 lines, the relative expression of *TrHSFB2a* was analyzed in transgenic *Arabidopsis* (Figure 4g), and two lines highly expressing *TrHSFB2a* were selected for a comparative analysis along with the wild type and T-DNA lines of *Arabidopsis* gene *AtHSFB2a*, purchased from *Arabidopsis* share platform “https://www.arashare.cn/index/ (accessed 18 December 2020)”, in which *TrHSFB2a* has been silenced. Under normal conditions, there was no significant difference in plant root length among groups after 7 d of transplanting in ½ MS medium (Figure 4d,e,h). However, the soil-grown overexpression *Arabidopsis* were significantly lower in fresh and dry weight compared to wild-type *Arabidopsis*, and T-DNA lines of *Arabidopsis* were significantly higher in fresh and dry weights compared to wild type (Figure 4i,j), suggesting that *TrHSFB2a* produced negative effects on early plant growth and development, which was also obvious in phenotypic observations (Figure 4f), suggesting that overexpression or silencing of *TrHSFB2a* at least do not cause detrimental effects on growth and developments of *Arabidopsis*. Two overexpression lines (OE3 and OE4), two T-DNA lines (denoted as T77 and T95) and the wild type (Wt) were subjected to different drought treatments.

**Figure 5:**
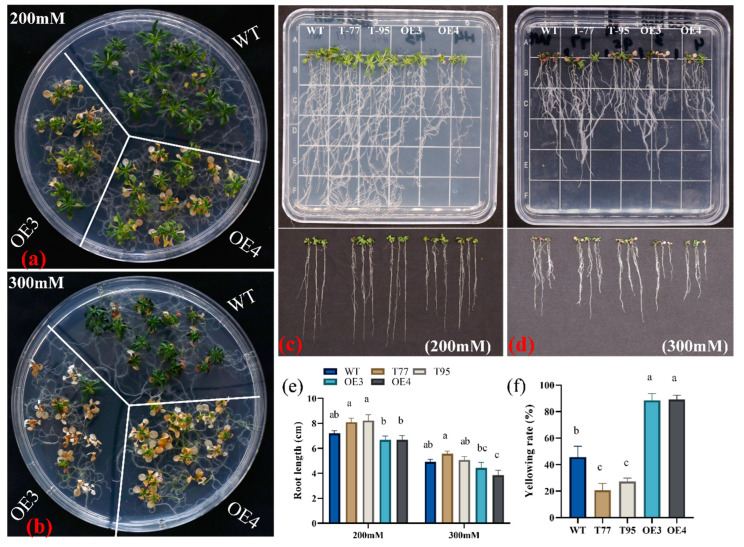
Comparison of drought-stress responses of overexpressing *TrHSFB2a* lines and RNA interference T-DNA lines and wild type grown on 1/2MS medium (with 3% sucrose + 0.7% agar) supplemented with 200 or 300 mM mannitol. (**a**,**b**) Display overexpression *TrHSFB2a Arabidopsis thaliana* along with wild type grown on 1/2MS medium supplemented with 200 and 300 mM mannitol, respectively, (**c**,**d**) show plant with roots growth on 1/2MS medium supplemented with 200 and 300 mM mannitol, respectively. (**e**) Root length on 1/2MS medium supplemented with 200 and 300 mM mannitol, respectively. (**f**) Plant-yellowing rate on 1/2MS medium supplemented with 200 mM mannitol. Bars represent mean ± SD values, and different small letters (a, b, and c) represent significant statistical difference among means (one-way ANOVA, followed by Tukey test) between different lines.

**Figure 6:**
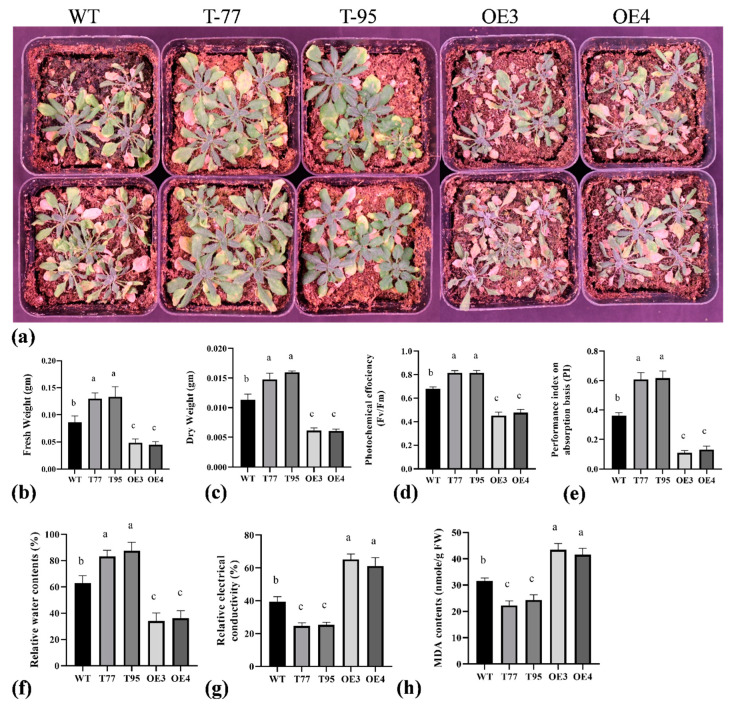
Comparison of drought stress tolerance of overexpressing *TrHSFB2a* lines and RNA interference T-DNA lines with wild type grown in nutrient soil by withholding water of 25 days old plants for up to two weeks. (**a**) Show phenotypes of wild, T-DNA lines and overexpression lines at 12 d of water withholding; (**b**–**h**) display a graphical representation of different phenotypic and physiological indicators of stress tolerance. (**b**) fresh weight; (**c**) dry weight; (**d**) photosynthetic efficiency (Fv/Fm); (**e**) performance index on absorption basis; (**f**) relative water contents (%); (**g**) Relative electrical conductivity (%); (**h**) MDA contents. Different alphabetic characters on bars represent statistically significant differences among variables.

Initially, 5-day-old seedlings of *Arabidopsis* (wild type and transgenic) were transferred to ½ MS plates containing 200 or 300 mM mannitol. After two weeks, the overexpression lines (OE-3 and OE-4) under 200 mM mannitol showed a significantly higher leaf-yellowing rate compared to the wild type, while most of the plants of overexpression lines under 300 mM mannitol dried; the wild type although had curled leaves, all were green (Figure 5a,b). Moreover, root length was significantly higher in T-DNA lines and was significantly lower in overexpression lines under 200 mM mannitol stress, indicating that *TrHSFB2a* possibly negatively regulates drought stress (Figure 5c–e). Three-week-old wild-type and transgenic *Arabidopsis* plants were also subjected to drought stress in nutrient soil by withholding water. Plants were well watered, and then, irrigation was stopped. After 12 days of water withholding, all the leaves of overexpression lines OE3 and OE4 wilted and dried, while wild-type plants showed less wilting compared to the overexpression lines, while T-DNA lines did not exhibit leaf wilting at this stage (Figure 6a). Three days after rewatering, more than 70% of wild-type plants recovered, while only about 30% of overexpression plants could survive. These observations indicated that the overexpression of *TrHSFB2a* significantly reduced drought tolerance, while suppressing its expression significantly increased drought tolerance in transgenic *Arabidopsis* at the seedling stage. Fresh weight, dry weight and photochemical efficiency determine how plants grow under stressed conditions. Relative water contents (RWC) of the plant showed their water status in terms of physiological consequences under water-deficient conditions. The cell membrane permeability and lipid peroxidation are the general indicators of damage in plants caused by different stresses and are measured as REC of electrolyte leakage and malondialdehyde (MDA) contents, respectively. Lipid peroxidation is generally associated with the oxidation of lipids of the cell membrane; thus, its quantification determines the extent of cell membrane damage. In the current study, the phytochemical efficiency of photosystem II and performance index was determined after 10 days of water withholding. Results showed that overexpression lines had significantly reduced photochemical efficiency of Photosystem II and the performance index compared to wild type, while T-DNA lines had significantly higher photochemical efficiency of photosystem II and performance index than that of wild type. Similarly, fresh weight, dry weight and RWC were significantly lower in overexpression lines compared to wild type and were significantly higher in T-DNA lines compared to wild type (Figure 6). In contrast, REC and MDA contents were significantly higher in overexpressed lines than that of wild type and significantly lower in T-DNA lines than that of wild type (Figure 6). These results, along with phenotypic data, demonstrated that *TrHSFB2a* negatively regulates drought stress, and RNA interference in T-DNA lines improved the drought tolerance in *Arabidopsis*.

### 2.5. TrHSFB2a Negatively Regulates the Heat Tolerance in Arabidopsis

**Figure 7:**
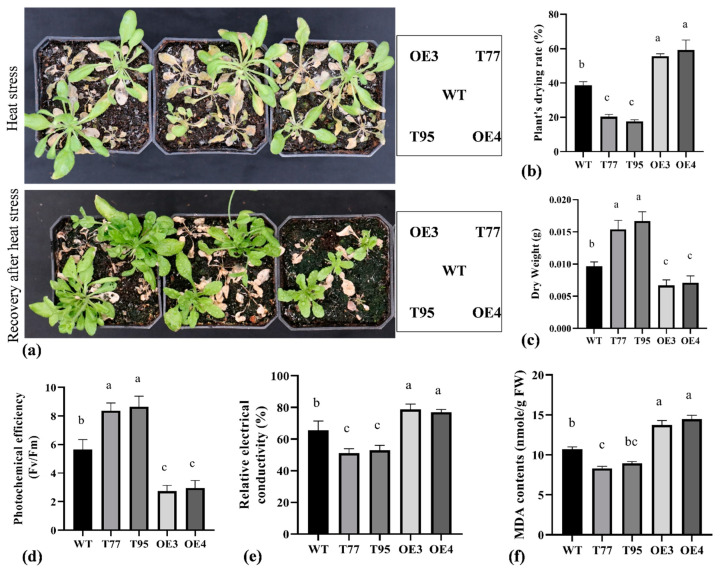
Comparison of heat stress tolerance of overexpressing *TrHSFB2a* lines and RNA interference T-DNA lines with wild type grown in nutrient soil (peat moss, vermiculite and perlite, 3:1:1) for 25 days and transferred to an incubator adjusted with 35 °C temperature. (**a**) Display *Arabidopsis* phenotypes after high-temperature treatment and recovery of plants after transferring to normal growth conditions. (**b**–**f**) display graphical representations of plants drying rate (%), dry weight (g), photochemical efficiency (Fv/Fm), relative electrical conductivity (%) and MDA contents (nmole/g FW), respectively. Bars represent mean ± SD values, and different small letters (a, b and c) represent significant statistical difference among means (one-way ANOVA, followed by Tukey test) between different lines.

Heat shock proteins and transcription factors are generally involved in regulating heat stress responses. One-month-old soil-grown plants were transferred to an incubator adjusted to 35 °C temperature for two weeks in order to validate the role of *TrHSFB2a* in *Arabidopsis* under high-temperature stress. Photochemical efficiency, REC and MDA contents were determined after one week, while plants' dying rate and dry weight were measured after two weeks when phenotypic differences appeared significantly. As shown in Figure 7, around 60% of plants of overexpression lines completely dried and could not recover when transferred in normal conditions. The rate of drying was significantly higher in overexpression lines than that of wild type. In comparison, T-DNA lines showed a significantly lower drying rate compared to wild type (Figure 7a). Photochemical efficiency and the plant’s dry weight were significantly lower for overexpression lines compared to wild type and significantly higher for T-DNA lines. In contrast, overexpression lines showed significantly increased REC % and MDA contents than that of wild type, and T-DNA lines had significantly reduced REC % and MDA contents compared to wild types, indicating that RNA interference lines had lower damage at the cellular level compared to wild type, while overexpression lines showed opposite trends. Collectively, phenotypic and physiological data demonstrated that *TrHSFB2a* negatively regulates heat tolerance in *Arabidopsis* at the seedling stage.

### 2.6. TrHSFB2a Negatively Regulates Salt Tolerance in Arabidopsis

**Figure 8:**
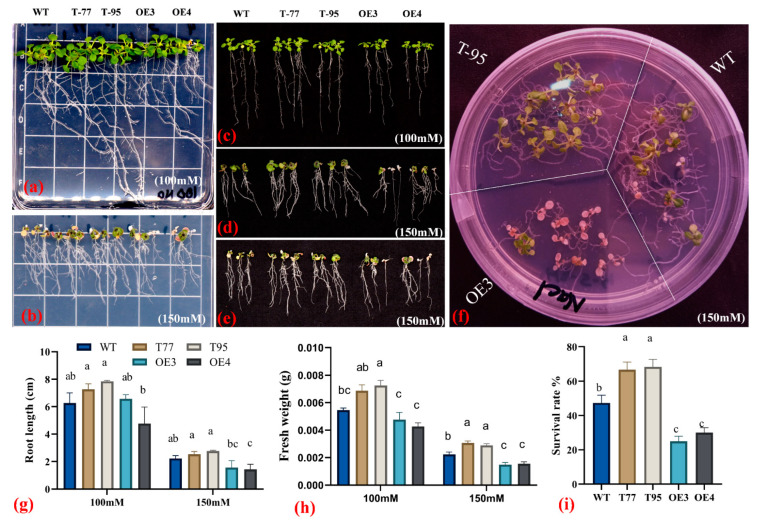
Comparison of salt stress tolerance of overexpressing *TrHSFB2a* lines and RNA interference T-DNA lines with wild type on 1/2MS medium supplemented with 100 or 150 mM NaCl. (**a**,**b**) show wild (WT), T-DNA lines (T-77 and T-95) and overexpression lines (OE3 and OE4) TrHSFB2a lines grown on 1/2MS medium supplemented with 100 mM and 150 mM NaCl, respectively, (**c**–**e**) show roots growth on 1/2MS medium supplemented with 100 mM and 150 mM NaCl, respectively; (**f**) plants survival/yellowing-related phenotypes on 1/2MS medium supplemented with 150 mM NaCl. (**g**–**i**) Show root length (cm), fresh weight (g) and survival rate (%) measured after 10 days of transplanting. Bars represent mean ± SD values determined from 30 seedlings, different small letters (a, b and c) represent significant statistical difference among means (one-way ANOVA, followed by Tukey test) between different lines within a treatment. Survival rate (%) was determined by counting survived and dead plants at 15 d of transplanting.

**Figure 9:**
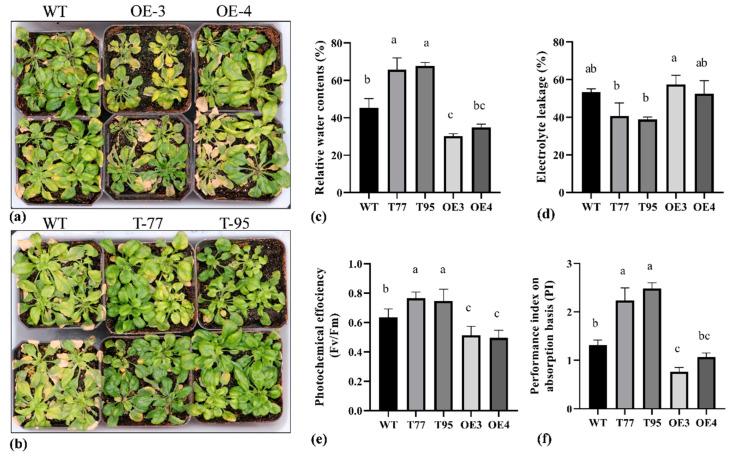
Comparison of salt stress tolerance of overexpressing *TrHSFB2a* lines (OE3 and OE4) and RNA interference T-DNA (T-77 and T-95) lines with wild type grown in nutrient soil for 28 days in normal conditions, irrigated with saline water gradients of 100 mM, 200 mM and 300 mM NaCl, each gradient for two times for two days. (**a**,**b**) Show phenotypic differences of wild + overexpression and wild + TDNA lines, respectively; (**c**–**f**) indicate relative water contents (%), electrolyte leakage (%), maximum quantum yield of PSII photochemistry (Fv/Fm) and performance index on absorption basis (PI_ABS_), respectively, under salt stress condition. Bars represent mean ± SD values, and different small letters (a, b and c) represent significant statistical difference among means (one-way ANOVA, followed by Tuky test) between different lines.

To check how the *TrHSFB2a* gene functions under salt stress conditions, we observed the overexpression and RNA interference T-DNA lines of *Arabidopsis* in ½ MS media and in soil. At five days old, seedlings were shifted to 1/2MS+Agar plates, supplemented with 100 mM or 150 mM NaCl, and phenotypic data were recorded at 10 d of transplanting. The results showed that *TrHSFB2a* had a significantly lower rate of survival (around 20%) compared to wild type (47.33%), and RNA interference T-DNA lines showed a significantly higher survival rate (more than 65%) than that of wild type (Figure 8), suggesting that overexpression of *TrHSFB2a* significantly reduced tolerance of salt, while RNA interference of *TrHSFB2a* can enhance salt tolerance of transformed *Arabidopsis* at the seedling stage. Root length was significantly higher in T-DNA lines compared to wild type under 100 mM and 150 mM salt stress, while one overexpression line (OE-3) showed significantly reduced roots length than that of wild type, and one (OE-3) did not show a significant difference with the wild type under both salt stress treatments. However, the total fresh weight was significantly higher in T-DNA lines and significantly lower in overexpression lines compared to wild type, respectively (Figure 8). Simultaneously, 10 d old wild and transgenic *Arabidopsis* lines grown on 1/2MS+Agar media were transferred into the soil, grown additionally for three weeks in nutrient soil, then irrigated with 100 mM, 200 mM and 300 mM NaCl gradients water, each twice after every two days (Figure 9a,b). RWC, EC, photochemical efficiency and performance index were measured after two times of 100 mM and 2 times 200 mM saline water irrigation, on the 8th d of treatment. Results showed that RWC, photochemical efficiency and performance index on an absorption basis were significantly lower in overexpression lines and were significantly higher in T-DNA lines compared to wild type, respectively. In contrast, EC % had significantly increased in overexpression lines and was significantly lower in T-DNA lines compared to wild type (Figure 9c–f). The data of physiological analysis, together with phenotypic observations, indicated that overexpression of *TrHSFB2a* reduced salt tolerance, while RNA interference for this gene can improve the salt tolerance of Arabidopsis at seedling and developing growth stages.

## 3. Discussion

Plant heat shock transcription factors (HSFs) play important roles in regulating biotic and abiotic stress responses, especially heat stress regulation [5,6]. HSFs can be divided into three classes (A, B and C) based on their domain characteristics, which have been reported to function differently in different stress responses [5]. The HSFs of class A have been generally reported as the positive regulator of stress tolerance in different plant species and possess an exclusive C terminal activation domain [16]. The class C HSFs have also been shown to play important functions in mediating the effects of heat, salt and osmotic stress in plants [37,38,39]. The class B HSFs including *HSFB1* and *HSFB2,* show repressive activity and are reported as negative regulators of stress tolerance in *Cicer arietinium* [40], *Oryza sativa* [41], *Glycine max* [42] and *Arabidopsis thialiana* [26,27]. In the current study, we reported and characterized an HSF of white clover *HSFB2a*, which belongs to the Class B HSFs family. Current study data suggested that the *TrHSFB2a* might negatively regulate heat, drought and salt tolerance in transgenic *Arabidopsis*.

### 3.1. Abiotic Stress Treatments Induce TrHSFB2a Expression

The expression pattern of *TrHSFB2a* was investigated under different treatments of abiotic stresses, including high temperature, PEG and salt stress. The expression of *TrHSFB2a* was strongly induced under drought stress, which is in accordance with our previous study of transcriptome analysis of white clover under drought stress [28]. Moreover, *TrHSFB2*a is strongly upregulated under heat stress like other HSFs members, as well as strongly expressed in response to NaCl stress. These observations are well in agreement with previous studies that HSFB2 candidates expressed under high temperature, high salinity and drought conditions [23,40,41]. Like stress-related cis-acting elements, including HSE, DRE, ABRE, MYCRS and MYBRS, present in the promoter region of the *HSFB2* genes in *Arabidopsis* and other plant species [5,43,44], they might also be involved in inducing response and the expression of *TrHSFB2a* in white clover under drought, heat and high salinity stresses, but it needs to be further verified.

### 3.2. TrHSFB2a Negatively Regulates Drought, Heat and Salt Stress in Arabidopsis

The overexpression of *TrHSFB2a* in *Arabidopsis,* although it enhanced the transcriptome abundance and RNA interference of *AtHSFB2a* in *Arabidopsis,* reduced the accumulation of *AtHSFB2a* transcripts. There were no significant differences in plant stature, root length or fresh and dry weights under normal conditions. It indicated that *TrHSFB2a* does not have significant effects on plant development under normal conditions, as similarly observed in the effects of HSBs of class B on other plant species [40,41]. However, under drought, salt and heat stress, the overexpression of *TrHSFB2a* significantly reduced plant growth compared to wild type, while RNA interference of *AtHSFB2*a significantly improved salt, heat and drought tolerance both in ½ MS media and in the soil. By increasing the stress levels (NaCl 150 mM and Mannitol 300 mM) or by extending stress duration, the overexpression of *TrHSFB2a* led to significantly higher drying and dying rates compared to wild type, while RNA interference of *AtHSFB2a* exhibited opposite trends. These observations agreeing has been observed in the transgenic lines developed by overexpression and RNA interference homolog of this *OsHSFB2b* in rice [41] and overexpression of *CarHSFB2b* of Chickpea in Arabidopsis [40].

RWC, photosynthetic activity, REC percentage and MDA contents are important indicators to assess the stress tolerance potential differences in plants. Under drought and heat stress treatment, the overexpression lines of *TrHSFB2a* showed higher REC % and MDA contents than that of wild type, and in T-DNA lines of *AtHSFB2a*, the accumulation of MDA contents and REC % was lower compared to wild type. As electrolyte leakage is an indirect indicator of cell membrane damage due to stress conditions [45,46], the higher REC in *TrHSFB2a* overexpression lines showed higher cell membrane damage, which had occurred there, while there was comparatively less cell membrane damage in T-DNA lines. One of the end products of lipid peroxidation due to external stresses is MDA, which is a result of membrane damage caused by free radicals [47,48], was higher in overexpression lines and lower in RNA interference lines compared to wild types, representing higher lipid damage in overexpression lines and a lower in T-DNA lines. Abiotic stresses on plants mainly reduce the photosynthetic efficiency of the plants under stress as a result of the negative effects of the abiotic stresses on the biosynthesis of photosynthetic pigments, photosystem performance, CO_2_ fixation and gases exchanges, and on carbohydrate metabolism, etc. The photosynthetic activity and photosystem performance index significantly reduced overexpression lines and significantly improved in T-DNA lines compared to wild-type user drought, salt and heat stress, indicating better adaptability of T-DNA line and reduced fitness by overexpressing *TrHSFB2a* gene in *Arabidopsis*. Moreover, lower RWC, as observed in overexpression lines, can reduce or even completely stop photosynthetic activity in a stressed condition. Leaf wilting, drying, RWC, photosynthetic system performance, REC and MDA-related to plant stress that has been already used to assess the function of HSFs under drought, salt and heat stress in transgenic plants [41,49,50]. These findings provide evidence that *TrHSFB2a* negatively regulates stress tolerance at least in *Arabidopsis*.

The HSFB class has been reported to act as a transcriptional repressor in plant species [23,26,41], including *Arabidopsis* [5,26]. In *Arabidopsis*, the single mutant *HSFB2B* and double mutant *HSFB1 HSFB2* induced the strong expression of stress defense-related genes, and thereby, significantly enhanced the resistance of *Arabidopsis* against pathogens [27]. These double mutants also improve heat tolerance in *Arabidopsis*, which might be because *HSFB1* and *HSFB2b* repress the expression of heat shock (HS) inducible genes required in the stress tolerance process [26]. Likewise, it is speculated that *TrHSFB2a* suppresses the expression of HS-inducible genes in white clover and as well as in overexpression *Arabidopsis* lines, while when it was mutated, or its expression decreased by RNA interference, it could not suppress the expression of HS-inducible genes, and full functionality of HS-inducible gens in stress condition in the absence of *AtHSFB2* protein might have improved the stress tolerance in T-DNA lines. The differences observed in the tolerance level of different stress between overexpression lines and T-DNA lines might be attributed due to the differential expression of HS-inducible genes because *TrHSFB2a* may repress the expression of such genes due to the existence of the core B3 repression domain (BRD) domain (Figure 1c). This domain is conserved in plant species and speculated as a core domain that repress the activation of downstream HSPs genes in different plant species [41], as observed in the case of *HSFB1* in Arabidopsis, but it needs to be tested.

In conclusion, the current study demonstrated that *TrHSFB2a* is expressed under various abiotic stresses, such as drought, heat and salt stress, and it negatively regulates drought, heat and salt stress, possibly by repressing the activation of HS-inducible genes under stressed conditions. Moreover, diminishing or reducing the expression of *TrHSFB2a* may have promising utility for enhancing the drought and heat tolerance of white clover.

## 4. Materials and Methods

### 4.1. Plant Growth Conditions

A total of 0.5 g of healthy and uniform-sized white clover (*Trifolium repens* L. cv. Landino) seeds were surface-sterilized with NaClO (0.5% (*w*/*v*), followed by being rinsed in autoclaved ddH_2_O 6 times and germinated in a plastic pot (24 cm length, 18 cm width and 9 cm deep) filled with sterilized moisturized quartz sand in a controlled growth chamber for seven days to maintain germination. Seven-day-old seedlings were irrigated with Hoagland’s nutrient solution for an additional three weeks (21 days) until the second leaves fully expanded, changing the Hoagland’s nutrient solution after every 2 days. The growth chamber was maintained at a 12 h photoperiod, with day/night temperatures of 23/19 °C, relative humidity of 75% and 250-μmol m^−2^⋅s^−1^ photosynthetic photon flux density. Four-week-old white clover plants were subjected to drought stress by 15% (*w/v*) polyethylene glycol (PEG) 6000, which was dissolved in Hoagland’s solution and samples were collected after 0 h, 1.5 h, 3 h, 6 h, 12 h and 24 h to isolate the total RNA for the subsequent cDNA library constructions. The total RNA was isolated from white clover plants (root + shoot) using an RNAprep Pure Plant Kit (TIANGEN Biotech (Beijing) Co., Ltd., Beijing, China) and was then reverse-transcribed to cDNA by an iScript^TM^ cDNA Synthesis Kit (Bio-Rad Laboratories, (Shanghai) Co., Ltd., Shanghai, China) according to the manufacturer’s manual instructions. *Arabidopsis thaliana* ecotype Col-0 was used for genetic transformation, and thus, grown as wild type. The *Arabidopsis* genotypes grown in the current stud consisted of wild-type (Col-0) and transgenic *Arabidopsis*, including overexpressing the *TrHSFB2* and RNA interference T-DNA lines of *AtHSFB2a* for the comparative study. The seeds of *Arabidopsis* were surface sterilized with alcohol (75% *w*/*v*) and (0.1% *w*/*v*) NaClO, and subsequently, propagated on ½ MS medium containing 3% sucrose and 0.7% agar, followed by being placed in the dark at 4 °C for 2–3 days for vernalization. Then, for germination, the seeds were transferred to the growth chamber in the set condition of 21 °C temperature with 65% relative humidity, 16 h photoperiod and 150-μmol m^−2^⋅s^−1^ photoactive radiation. After 5 days, the equal-sized seedlings were transferred to square plates containing 1/2MS medium with sucrose (3%) and agar (0.7%) and arranged at 90 degrees in the same growth chamber for observing roots and shoot growth differences under normal and stressed conditions between transgenic and wild type *Arabidopsis* plants. Moreover, after 10 days, well-grown uniform-sized wild and transgenic *Arabidopsis* plants were transferred into plastic pots (10 cm length, 10 cm width and 7 cm deep) filled with nutrient soil consisting of peat moss, vermiculite and perlite (3:1:1) and placed in the growth chamber for completing rest growth periods or being subjected to different stress treatments after about 25 d of transplanting.

### 4.2. TrHSFB2 Gene Isolation from White Clover and Bioinformatics Analysis

Initially, the homologous sequences of *TrHSFB2a* were obtained through the BLAST program of NCBI “http://www.ncbi.nlm.nih.gov/Blast (accessed on 12 April 2020)” [51] by searching and identifying conserved sequences region of *TrHSFB2a* with closely related species and then aligning these sequences with partially published white clover genomes on the NCBI database https://www.ncbi.nlm.nih.gov/genome/13404 (accessed on 12 April 2020) [52] to obtain gene sequence information for the designing primers. The premier primer-5 software was used to design primers for *TrHSFB2a* amplification, and thereby, obtained a full-length CDS sequence of *TrHSFB2a* from the cDNA library by using the primer pair *TrHSFB2*-F CTCGCGAACCTTCTAGAACTCTCA and *TrHSFB2*-R TCCCTAATCCATCTAACATCAGGTGTCA through the touch-down PCR method and Phanta Max Master Mix (see the procedure detail in Supplementary Methods and Supplementary Table S1). The full-length amplified PCR fragments were excised and cloned into the pMD19 simple vector for sequencing; after confirming the *TrHSFB2* sequence from three sequenced clones through a homology search on the NCBI database, an open reading frame of 924bp was cloned into the pBI21 overexpression vector (detail below). The molecular weight and pI values of primary *TrHSFB2* protein sequences were determined using the ExPASy software http://web.expasy.org/protparam (accessed on 1 March 2022) [53]. Clustal Omega “https://www.ebi.ac.uk/Tools/msa/clustalo/ (accessed on 1 March 2022)” [54] and PSORT “https://www.genscript.com/psort.html (accessed on 10 December 2020)” [55] were used for multiple sequence alignment and subcellular protein localization, respectively. The motif and conserved domains were identified using “https://www.ncbi.nlm.nih.gov/Structure/cdd/wrpsb.cgi (accessed on 1 March 2022)” [56], and open reading frames (ORFs) were predicted using the NCBI program https://www.ncbi.nlm.nih.gov/orffinder (accessed on 10 May 2020) [57]. The construction of the phylogenetic tree was carried out through the MEGA 11 bioinformatic tool.

### 4.3. Plasmid Construction, Genetic Transformation and Developing Homozygous Overexpression Lines of TrHSFB2a

pSUPER1300-GFP and pBI121 vectors were used for subcellular localization and for constructing the overexpression of *Arabidopsis,* respectively. The complete coding region (without stop codon) of *TrHSFB2a* was amplified from a previously constructed pMD19 simple vector by the PCR reaction using Prime STAR^®^ Max DNA-Polymerase (Takara-Biology Technology (Beijing) Co., Beijing, China) by following manual instructions, and inserted into the restriction sites of *XbaI* and *BamHI* of the linearized pBI121 (containing CaMV 35S promoter) and *XbaI* and *KpnI* site of the linearized pSuper1300-GFP using the EasyGeno Assembly Cloning kit (TIANGEN Biotech (Beijing) Co., Ltd., Beijing, China) to generate *TrHSFB2*::GFP in-frame fused protein according to manual’s instruction. The primers for amplifying the coding region and flanking vector sequences for both constructs are enlisted in Supplementary Table S1. The constructed pBI121 vector (CaMV 35S::TrHSFB2a) was then mobilized into the floral tissues of the *Arabidopsis thaliana* genotype “Col-0” by *Agrobacterium*-mediated transformation through the floral dip method [58]. The seeds of transformed plants were harvested, surface sterilized and sown on the selective media of ½ MS media with Kanamycine-50. Surviving plants were transferred into the soil at the four-leaf stage, confirmed by PCR for the presence of *TrHSFB2a*, and thereby developed ten independent transgenic overexpression lines. The expression level of *TrHSFB2a* in transgenic lines was identified by qRT-PCR and selected overexpression lines OE3 and OE4 and OE6 for further experiments based on relative expression levels. Two T-DNA lines (N675207 and N677095) of the *Arabidopsis* gene *AtHSFB2A* (Gene ID: At5g62020) were purchased from *Arabidopsis* mutants stock share platform AraShare “https://www.arashare.cn (accessed on 18 December 2020)” for the comparative functional analysis of *TrHAFB2a* in *Arabidopsis*.

### 4.4. Subcellular Localization

The fusion constructs *TrHSFB2*::GFP and control GFP vector were inserted into the *Agrobacterium* strain EHA105 competent cells using a standard protocol. Briefly, the transformed *Agrobacterium* culture was shaken for 2–3 h in YEB (Yeast Extract Beef) solution without an antibiotic at 28 °C, then centrifuged at 4000 rpm for one min (minute) and plated 100 µL on YEB medium containing rifamycin-20 and kanamycin-50, and incubated at 28 °C for 72 h followed by positive colonies selection using colony PCR. Cell culture was shaken at 28 °C until the OD-600 value reached 0.8, then the 30 mL cell culture was centrifuged at 4000 rpm for 10 min at 4 °C, dissolved in autoclaved water containing 5% sucrose and 0.02% silwet and, finally, introduced into tobacco leaves. Then the subcellular location of *TrHSFB2a* protein was determined by transiently expressing the *TrHSFB2a::GFP* fusion protein in the leaves of *Nicotiana benthamiana.* Tobacco plants were placed in a growth chamber in the dark, set at a day/night temperature of 23/19 °C, with 75% relative humidity for 24 h, followed by observing the GFP localization using a fluorescent microscope (Olympus).

### 4.5. Relative Expression of TrHSFB2a

To determine the relative expression level of *TrHSFB2a* in white clover under drought, heat and salt stress conditions, the four-week-old white clover plants were subjected to drought or salt stress by irrigating with Hoagland’s solution containing PEG6000 15% *w/v* or 200 mM L^−1^, respectively, in a controlled growth chamber. High-temperature stress was induced by placing the four-week-old white clover plants in a growth chamber adjusted with a continuous temperature of 35 °C. The processing times were 0 h, 1.5 h, 3 h, 6 h, 12 h and 24 h. The plant samples were collected, immediately frozen in liquid nitrogen, and stored at −80 °C for the subsequent process of total RNA extraction. Plant tissues (leaf, stem and roots) were ground in liquid nitrogen and 100 mg of tissue powder was used to extract the total RNA using HiPure Universal RNA Mini Kit (Magen, Beijing) by following the manual’s instructions. After confirming the quantity and quality on the NanDrop spectrophotometer and the RNA integrity on 1.5 agarose gel, cDNA was synthesized by reverse transcription of the first strand of RNA using MonScript™ RTIII All-in-One Mix with dsDNase (Monad Biotech Co., Ltd., Beijing, China) following kit protocols. Later, the relative gene expression was determined using 2X M5 HiPer SYBR Premix EsTaq (with Tli RNaseH) (Mei5 Biotechnology, Co., Ltd., Beijing, China) and the CFX96 Real-Time PCR detection system (Bio-RAD) according to manual instructions. The qRT-PCR primers are provided in Supplementary Table S1. The TrB-Actin gene was used as an internal control for white clover. The amplification conditions were as follows: initial denaturation at 95 °C for 5 min, then, 40 cycles at 95 °C for 10 sec and 60 °C for 30 s. The relative difference in the expression of the genes was calculated by the 2^−^^ΔΔCt^ method [59].

For determining *TrHSFB2a* expressions in transgenic *Arabidopsis*, the seeds of 10 overexpression lines were sown on ½ MS media supplemented with 3% sucrose and 0.7% agar, followed by vernalization for 2 days. After germination, seedlings were grown for an additional 10 days in the same ½ MS media plates placed in a growth chamber set at 21 °C with 65% relative humidity, 16 h photoperiod, and 150 μmol m^−2^⋅s^−1^ photoactive radiation. Then, the extraction of the total RNA, reverse transcription and qRT-PCR analysis was the same as stated above for white clover.

### 4.6. Drought, Salt and Heat Stress in Arabidopsis

The drought, salt and heat stress treatments were according to Jia et al. (2021) [60]. Briefly, the seeds of homozygous T3 lines of transgenic Arabidopsis (overexpression and T-DNA lines) were used for studying stress responses. For drought and salt stress treatments in ½ MS medium, 5 d old wild and transgenic lines were transferred into ½ MS medium (15 plants of each genotype with three technical repeats) supplemented with 200 mML^−1^ and 300 mML^−1^ of mannitol or 100 mML^−1^ and 150 mML^−1^ of sodium chloride (NaCl). The phenotypes of plants under stress were observed daily for up to 26 days. Photographs of the plants were taken using a digital camera. The yellowing rate (senescence) was calculated by counting the respective plant at the end of the experiment from each treatment. One-month-old wild and transgenic lines were also subjected to drought, heat and salt treatment in nutrient soil (*peat moss*, *vermiculite*, *perlite*, 3:1:1) in controlled growth chamber conditions, as stated above. Drought stress treatment was induced by withholding the irrigation for 22 days. Salt treatment was applied by irrigation with 100 mML^−1^, 200 mML^−1^ and 300 mML^−1^ of NaCl every two times after every two consecutive days. For inducing heat stress, the one-month-old soil-grown WT and transgenic plants were transferred to another growth chamber adjusted with a continuous temperature of 35 °C for two weeks.

### 4.7. Determination of Relative Water Contents (RWC)

RWC was determined by following Barrs and Weatherley [61]. A total of 0.3 g of leaf blade samples were collected between 9:00–10:00 am, wrapped well in ordinary absorbent paper and placed into centrifuge tubes of 50 mL, followed by filling the tubes with water, covered with a lid and placing in a protected place for 24 h. When the leaves had absorbed water at full saturation level, we took out the leaf samples, wiped off the surface water, weighed the saturated fresh weight, and placed the samples in a blast oven set at 105 °C temperature for 45 min. Finally, we dried the samples at 75 °C to reach a constant weight and measured dry weights. Three biological repeats were used for all treatments. RWC of the leaves were calculated by employing the following formula:

RWC % = [(FW − DW)/(TW − DW)] × 100

FW, TW and DW represent fresh weight, saturated fresh weight after drenching the leaves in water for 12 h and dry weight, respectively.

### 4.8. Determination of Relative Electrical Conductivity (EC)

Relative electrical conductivity was determined by following Blum and Ebercon [62]. Briefly, 0.1 g of leaf blade samples of the different materials in the study were placed into test tubes filled with 15 mL of deionized water. The initial conductivity (C_initial_) was determined by the conductivity meter (YSI Model 32) after 24 h, then we boiled the samples at 100 centigrade for 15 min, followed by cooling down at room temperature and measuring their final conductivity (C_max_). Three biological repeats were used for all treatments. The following formula was used to calculate EL:

Relative electrical conductivity EL = C_initial_/C_max_ × 100

where C_initial_ is initial conductivity and C_max_ is final conductivity.

### 4.9. Determination of Chlorophyll Fluorescence Parameters

Chlorophyll fluorescence parameters were measured according to Jia et al. (2021) [60]. Briefly, for determining chlorophyll fluorescence parameters, we placed the soil-grown WT and transgenic lines in the dark for 30 min, and measured the maximum quantum yield of PSII photochemistry (Fv/Fm) and PI_ABS_ by a pulse-amplitude modulation portable chlorophyll fluorometer (PAM-2500), using 15 replicates for each treatment.

### 4.10. Determination of Malondialdehyde (MDA) Content

The MDA contents of the leaves of different materials in the study were determined by following Heath and Packer (1968) method. Briefly, 0.1 g leaf tissues were immediately frozen in liquid nitrogen, followed by thoroughly grounded in ice within 2 mL of 50 mM pre-cooled phosphate-buffered saline (PBS) solution (pH 7.8) and centrifuged at 12,000× *g* for up to 30 min at 4 °C. The supernatant was taken and used to extract and determine MDA content by adding 1 mL of reaction solution comprising of trichloroacetic acid (20% *w/v*) and thiobarbituric acid (0.5% *w/v*) of a 0.5 mL solution of crude enzyme. Then samples were heated at 95 degrees Celsius for up to 30 min in a water bath, followed by quickly cooling to room temperature in an ice bath, with continuous gentle shaking to avoid bubble formations, and then centrifuged at 10,000× *g* for 10 min, removing the air bubbles, if any, with the pipette. Finally, we took out the supernatant and measured absorbance values at the wavelength of 532 nm and 600 nm. Three biological repeats were used for all treatments. To calculate MDA contents values, we subtracted the absorbance value of 600 nm wavelength from the absorbance value obtained at 532 nm wavelength by using an extinction coefficient of 155 mM^−1^ em^−1^ [63].

MDA (nmole g^−1^ DW) = (A532 − A600) × V × 1000/155 × W

where A532 is the absorbance at 532 nm, A600 is the absorbance at 600 nm, V is the extraction volume and W is the dry weight of the leaf.

### 4.11. Statistical Analysis

Statistical analyses were performed using IBM SPSS and GraphPad Prism 8.3.0 software, and graphs were made using GraphPad Prism 8.3.0 software.

## Data Availability

The data presented in this study are available on request from the corresponding author.

## Supplementary Materials

The supporting information can be downloaded at: https://www.mdpi.com/article/10.3390/ijms232112769/s1.
