# Supplementary material for: A Heat Shock Transcription Factor TrHSFB2a of White Clover Negatively Regulates Drought, Heat and Salt Stress Tolerance in Transgenic Arabidopsis
Source: Int J Mol Sci. 2022 Oct 23;23(21):12769. doi: 10.3390/ijms232112769 (PMC9654841; doi:10.3390/ijms232112769)
Supplement: Supplementary file 1 [file ijms-23-12769-s001.zip › ijms-1852470-supplementary.pdf]

Supplementary Methods: For amplifying the *TrHSFB2a* gene from white clover, 25 days old white clover plants were treated with PEG6000, and total RNA was isolated from the leaf samples collected after 0h. 1.5h, 3h, 6h, and 12h. After making the cDNA library of all samples, the cDNA of all samples was mixed together for increasing the probability of amplifying the *TrHSFB2a*. With primer pairs *TrHSFB2*-F CTCGCGAACCTTCTAGAACTCTCA and *TrHSFB2*-R TCCCTAATCCATCTAACATCAGGTGTCA and using 2 x Phanta Max Master Mix (Vazyme Biotech Co., Ltd., China) according to manual instruction. The specific PCR conditions were; initial denaturation at 95 °C for 3 min, then 20 cycles with denaturation at 95 °C for 15 sec, annealing at 56.5 – 66.5 for 15 sec by reducing 1 °C after every two cycles, extension at 72 °C for 1 min, and then 20 cycles with denaturation at 95 °C for 15 sec, annealing at 56.5 °C for 15 sec, extension at 72 °C for 1 min and then final extension at 72 °C for 5 mints. PCR products were isolated on 1.5% Agarose gel, excised the band, and cloned in an easy vector pMD19 (Takara Bio Inc., Beijing) by following manufacturer,s manual protocol for confirming gene sequence and saving for late use. Constructed plasmids were saved 20 °C for short time and -80 °C for longtime storage.

Supplementary Table S1. Primers for PCR and qRT-PCR.

TrHSFB2-F	5'- CTCGCGAACCTTCTAGAACTCTCA-3'
TrHSFB2-R	5'-TCCCTAATCCATCTAACATCAGGTGTCA-3'
pBI121-Oligo 1 (*XbaI*) -F	5'-CACGGGGGACTCTAGAATGGCTCCGTCGGCGGAA-3'
pBI121-Oligo 2 (*BamHI*) -R	5'-GACCACCCGGGGATCCATTACATACGCTCTTATTTCTCT-3'
pSUPER1300- Oligo 1 (*XbaI*) -F	5'-CACGGGGGACTCTAGAATGGCTCCGTCGGCGGAA-3'
pSUPER1300- Oligo 2 (*KpnI*) - R	5'-GACCACCCGGGGATCCATTACATACGCTCTTATTTCTCT-3'
TrHSFB2 qRT-PCR-F	5'- GGCTTCAAATGCAACAGTGG-3'
TrHSFB qRT-PCR-R	5'- TTTCTCAGCTTCTCGTTCTCG-3'
Trβ-Actin101 qRT-PCR-F (White clover)	5'-TGCTTGATTCCGGTGATGGTGTG-3'
Trβ-Actin101 qRT-PCR-R (White clover)	5'-TTCTCGGCAGAGGTACTGAAGGAG-3'
Atβ-Actin2 qRT-PCR-F (Arabidopsis)	5'-CCATCCTCCGTCTTGACCTT-3'
Atβ-Actin2 qRT-PCR-R (*Arabidopsis*)	5'-ACTTGCCCATCGGGTAATTC-3'

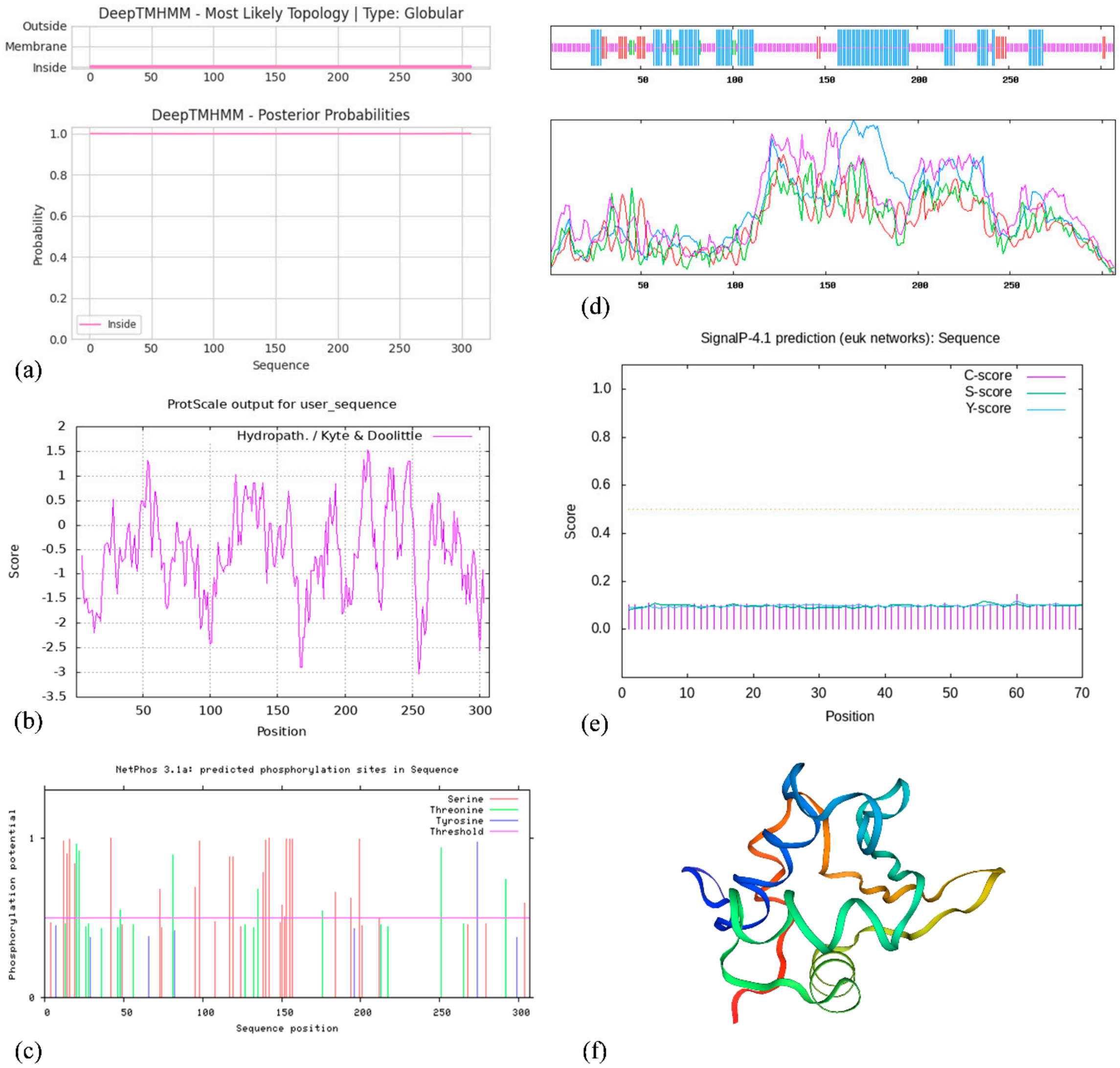

Supplementary Figure S1. Bioinformatics analysis of TrHSFB2a protein

(a) Transmembrane domain prediction of antiporter protein of *TrHSFB2a*, **(b)** Hydrophobicity analysis of TrHSFB2a, **(c)** Predicted phosphorylation sites of *TrHSFB2a*, **(d)** Secondary structure prediction of *TrHSFB2a* determined by SOPMA (Alpha helix blue, extended strand red, beta turn green, and random coil yellow), **(e)** Signal peptide of *TrHSFB2a* **(f)** Protein structure/model of *TrHSFB2a*
